# Systematic Review and Meta-Analysis of Oxidative Stress and Antioxidant Markers in Oral Lichen Planus

**DOI:** 10.1155/2021/9914652

**Published:** 2021-09-27

**Authors:** Jia Wang, Jingjing Yang, Chen Wang, Zhibai Zhao, Yuan Fan

**Affiliations:** ^1^Department of Oral Medicine, The Affiliated Stomatological Hospital of Nanjing Medical University, Nanjing, 210029 Jiangsu, China; ^2^Jiangsu Province Key Laboratory of Oral Diseases, Nanjing, 210029 Jiangsu, China; ^3^Jiangsu Province Engineering Research Center of Stomatological Translational Medicine, Nanjing, 210029 Jiangsu, China; ^4^College & Hospital of Stomatology, Anhui Medical University, Key Laboratory of Oral Diseases Research of Anhui Province, Hefei 230032, China

## Abstract

**Background:**

Oral lichen planus (OLP) is a relatively common chronic inflammatory disease of unknown etiology, which might be caused by oxidative stress and impaired antioxidant defense.

**Objective:**

To systematically investigate the markers of oxidative stress and antioxidant systems in the saliva and blood from OLP patients and healthy controls.

**Methods:**

The PubMed, Cochrane Library, and Embase were systematically queried to collect data from studies in which oxidative stress/antioxidant markers from OLP and healthy subjects had been evaluated until March 10, 2021.

**Results:**

A total of 28 studies fulfilled inclusion criteria, and 25 of them, having 849 OLP patients and 1,052 control subjects and analyzing 12 oxidative stress and antioxidant state marker levels, were subjected to meta-analysis. We found a significant decrease in total antioxidant capacity (TAC) and uric acid (UA) and a significant increase in malondialdehyde (MDA) and nitric oxide (NO) levels in the saliva and serum/plasma of OLP patients. Moreover, a significant elevation of 8-hydroxy-deoxyguanosine (8-OHdG) and advanced oxidation protein product (AOOP) level and a decrease in vitamin C were also observed in the saliva of the OLP group. In contrast, salivary vitamin A, zinc, glutathione peroxidase (GPx), vitamin E, and nitrite were not significantly different between the two groups. In single studies, markers of oxidative stresses such as superoxide dismutase (SOD) and 8-isoprostanelevels were elevated in OLP, and antioxidant parameters such as glutathione (GSH) and total protein (TP) levels were dysregulated.

**Conclusion:**

This meta-analysis helps to clarify the profile of oxidative stress and antioxidant state markers in OLP patients although existing evidence is rather heterogeneous and many studies are affected by several limitations. Larger and more standardized studies are warranted to ascertain whether these markers are potential causes or effects of OLP and whether antioxidant therapy improving oxidative stress will be useful.

## 1. Introduction

Oral lichen planus (OLP), a chronic inflammatory disease characterized by relapses and remissions, could be found in about 2% of people all around the world, with an overall age-standardized global prevalence of 1.27% (0.96% in men and 1.57% in women) in the general population [1], more commonly found in women aged between 50 and 60 years [2]. OLP is a precancerous lesion, and a malignant transformation varies between 0% and 12.5% [3]. Although its etiology is unknown, it is believed to be mediated by an immune disorder [4]. Therefore, more extensive knowledge and more accurate diagnostic tools of OLP are particularly important.

Recent years have seen increasing research interest in oxidative stress (OS) in the pathogenesis of several diseases, such as inflammatory, chronic degenerative (Alzheimer's disease), cardiovascular, or autoimmune [5–8]. OS modifies the normal intracellular balance, producing excessive oxidant substances, i.e., reactive oxygen species (ROS) and reactive nitrogen species (RNS) and resulting in a relative deficiency of enzymatic and nonenzymatic antioxidants [9, 10]. The members of antioxidant systems are currently used as “indirect” biomarkers of oxidative stress generation [11]. Enzymatic antioxidant systems comprise superoxide dismutase (SOD), catalase (CAT), and glutathione peroxidase (GSHPx) [12, 13]. Nonenzymatic antioxidant systems include minerals and vitamins [14].

OS may play a central role in the etiology of OLP [15]. The subepithelial infiltration of T lymphocytes in OLP contributes to the local production of cytokines, which can stimulate production of ROS [16]. The presence of apoptosis is a hallmark feature in OLP. ROS are essential mediators of apoptosis and may result in the dysfunction of keratinocytes and their impaired apoptosis [17].

A recent study provided a complete review of the association of micronutrients with OLP, suggesting a decrease in antioxidant levels and an increase in oxidant levels in OLP patients [18], both being significant. In line with these findings, increased nitric oxide (NO) and malondialdehyde (MDA) have also been found in patients with OLP and suggested as biomarkers for monitoring disease activity and therapeutic responses in OLP [19, 20]. Overall, these studies show that OLP patients are more susceptible to oxidant-antioxidant imbalance. To date, however, there is no consensus on the most appropriate biomarkers of oxidative stress and antioxidant systems in OLP. No quantitative systematic meta-analysis has been conducted on this subject either. Therefore, we aimed to perform a meta-analysis to quantify the association between OLP and these markers.

## 2. Methods

### 2.1. Protocol and Registration, Search Strategy, and Eligibility Criteria

The protocol was registered at PROSPERO (CRD42020199851) in accordance with the Cochrane Handbook [21] and PRISMA guidelines [22]. The review focused on the alterations of oxidative stress and antioxidant barrier markers in the saliva or serum with and without OLP.

We conducted an electronic literature search using PubMed, Cochrane Library, and Embase until 03/10/2021 for studies comparing oxidative stress and antioxidant markers between OLP patients and healthy controls. Controlled vocabulary terms (MeSH) and the following keywords were used in the search strategy: (lichen planus, oral [MeSH Terms]OR oral lichen planus OR OLP) AND (((ROS OR reactive oxygen species OR reactive nitrogen species OR free radicals OR oxidized LDL OR protein carbonylation OR lipid peroxidation OR MDA OR malondialdehyde OR thiobarbituric acid reactive substances OR asymmetric dimethylarginine OR 8-OHdG OR 8-hydroxy-guanosine OR homocysteine OR SOD OR superoxide dismutase OR glutathione OR GPX OR glutathione peroxidase OR glutathione reductase OR catalase OR vitamin A OR vitamin C OR vitamin E OR a-carotene OR b-carotene OR ascorbic acid OR paraoxonase OR albumin OR uric acid OR high-density lipoprotein cholesterol OR zinc OR NO OR nitric oxide OR nitrite OR peroxide OR 8-F2-isoprostane OR protein carbonyl) OR (antioxidants [MeSH Terms] OR anti-oxidants OR anti oxidants OR anti-oxidant effect OR anti oxidant effect OR anti-oxidant effects OR anti oxidant effects OR total antioxidant∗ OR antioxidative stress OR antioxidant∗ OR antinitrative stress)) OR (oxidative stress [MeSH Terms] OR oxidative stresses OR stresses, oxidative OR stress, oxidative OR oxidation OR oxidative damage OR nitrative damage OR oxidant∗ OR total oxidant∗)). The search was not limited to specific years. An English language restriction was imposed. Gray literature was searched in OpenGrey.

The inclusion criteria were as follows: (a) population—patients were clinically and histopathologically diagnosed with oral lichen planus; (b) exposure—at least one oxidative stress or antioxidant marker was measured in the saliva or serum/plasma sample; (c) comparators—the article included both the healthy control group and OLP group; (d) outcomes—sufficient information regarding the levels of oxidative stress and antioxidant system markers was presented for further analysis; and (e) study designs—the research was designed as either a case-control study or a cross-sectional survey.

### 2.2. Data Collection and Quality Assessment

After independent searches, titles and abstracts were reviewed by 2 reviewers (J.W. and JJ. Y) who then read the full texts to determine the final eligibility. Discrepancies were resolved through consensus or involvement of a third author (Y.F.). One author (J.W.) extracted all the data, which were independently verified by another author (JJ. Y). For studies that did not provide specific data, we contacted the authors to request their original data. If no response was received, the reviewers excluded their studies. Data extracted from each eligible study included the following: (a) article information—first author, year, country, journal, title, study design, and sample size; (b) participant characteristics—age, sex, and types of OLP; and (c) outcomes—mean ± SD or median (IQR), median (min–max) of OS and antioxidant biomarker levels (for both OLP patients and controls), and sample source.

Quality scores of case-control (CC) studies were assessed with the Newcastle-Ottawa scale (NOS), which was based on 3 topics: selection, comparability, and exposures (11 items in total, one star for each) [23]. Studies with 7 to 9 stars were recognized having high quality, those with 5 to 6 stars as medium quality, and those with <5 stars as low quality. The quality of cross-sectional (CS) survey was evaluated by the Agency for Healthcare Research and Quality (AHRQ) (one point for each) [24]. The qualities of studies with 0–3 points were recognized as low, those with 4–7 points as moderate, and those with 8–11 points as high. The Review Manager 5.4 special software for the Cochrane collaborative network was used to output the result of NOS and AHRQ.

### 2.3. Statistical Analysis

Once more than two studies reported a similar biomarker, we would conduct meta-analysis. The sample size and mean ± standard deviation (SD) were used to generate the effective size. The standardized mean difference (SMD) was calculated because of some clinical and methodological heterogeneity between studies. The 95% confidence interval (95% CI) was used to estimate the statistical significance of the pooled effective size. *P* < 0.05 was considered statically significant. To compare parallel studies, a method described by Hozo et al. [25] was used to calculate an estimate of the correspondent mean and SD if the data were reported as median (IQR) or median (min–max). We assessed statistical heterogeneity using the *I*^2^ statistic. When *I*^2^ > 50%, we used a random effects model to calculate effect size; otherwise, the fixed effects model was used [26]. Values of 25%, 50%, and 75% indicate low, moderate, and high heterogeneity, respectively.

When the number of included articles was more than 6 and *I*^2^ > 50%, we further conducted subgroup analysis to explore possible explanation for heterogeneity and sensitivity analysis to confirm the robustness of the main findings. A subgroup analysis was done as categorized by race (Asian vs. European), and the sensitivity analysis was performed by removing the studies one by one. If the number of included studies was smaller than 10, visual analysis of funnel plots was not very reliable [27]. All data were analyzed using Review Manager 5.4 (the Nordic Cochrane Center, the Cochrane Collaboration, Copenhagen, Denmark).

## 3. Results

### 3.1. Literature Searches

**Figure 1:**
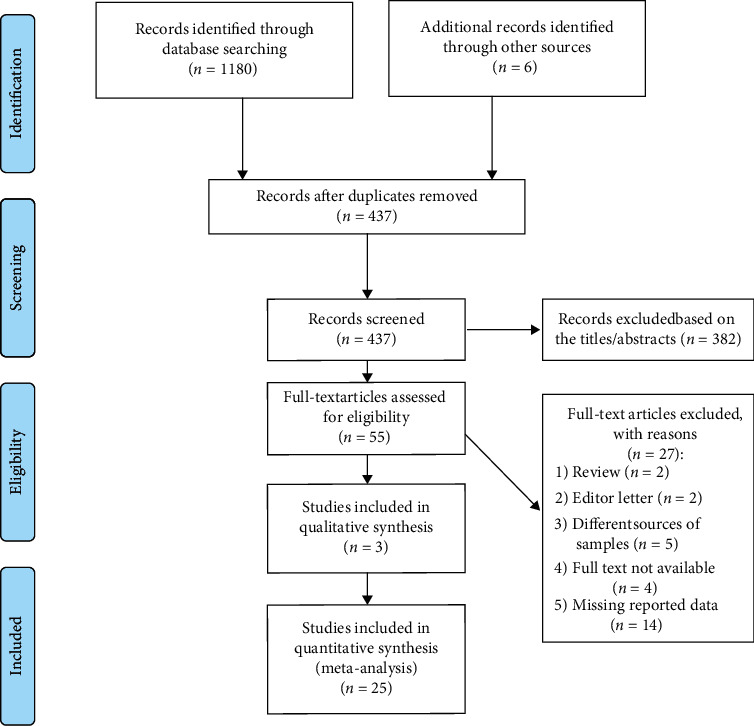
PRISMA flow diagram of the study selection process.

The flow diagram (Figure 1) presented the review selection process and the study selection process. We retrieved 1,186 records published prior to 03/10/2021 from the databases. After excluding duplicates, 437 records were filtered based on title and abstract. The full text of 55 of these records was retrieved for further assessment. After the full texts were read, 27 articles were excluded. In total, 28 articles fulfilled the inclusion criteria and 25 studies were eligible for our meta-analysis.

### 3.2. Study Characteristics

**Figure 2:**
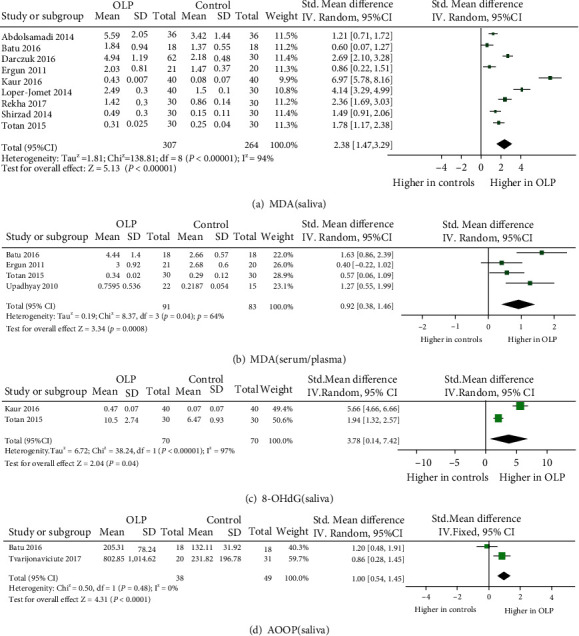
Evidence of increased oxidative stress markers in oral lichen planus (OLP). Forest plots show higher malondialdehyde (MDA) in saliva and serum/plasma and 8-hydroxy-deoxyguanosine (8-OHdG) and advanced oxidation protein product (AOOP) level in saliva from subjects with OLP compared to healthy controls (*P* < 0.05 for each).

**Figure 3:**
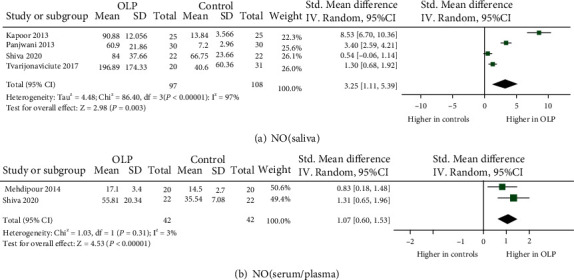
Evidence of increased nitrosative stress markers in oral lichen planus (OLP). Forest plots show higher nitric oxide (NO) levels in saliva and serum/plasma from OLP patients compared to healthy controls (*P* < 0.05 for each).

**Figure 4:**
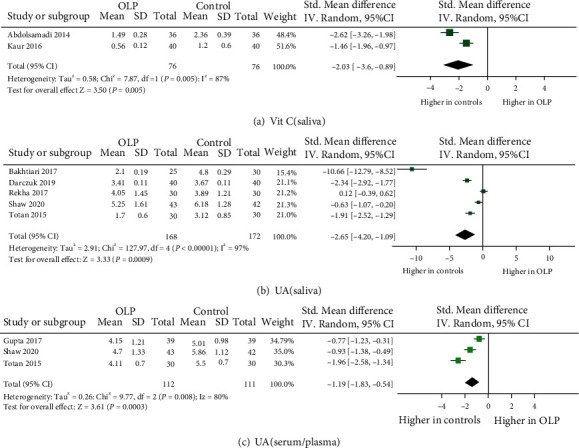
Evidence of decreased antioxidant defenses in oral lichen planus (OLP). Forest plots show lower levels of uric acid (UA) in saliva and plasma/serum and vitamin C in saliva from OLP patients to healthy controls (*P* < 0.05 for each).

**Figure 5:**
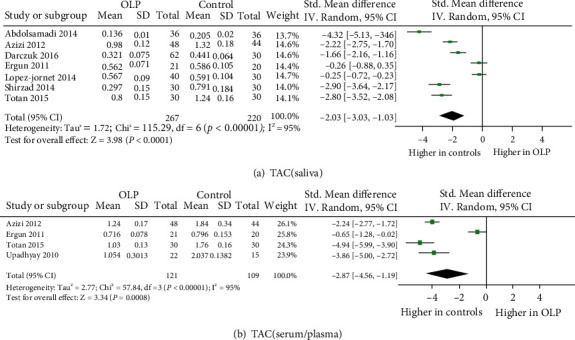
Evidence of decreased total redox status in oral lichen planus (OLP). Forest plots show lower levels of total antioxidative capacity (TAC) in saliva and plasma/serum from OLP patients than from healthy individuals (*P* < 0.05 for each).

**Figure 6:**
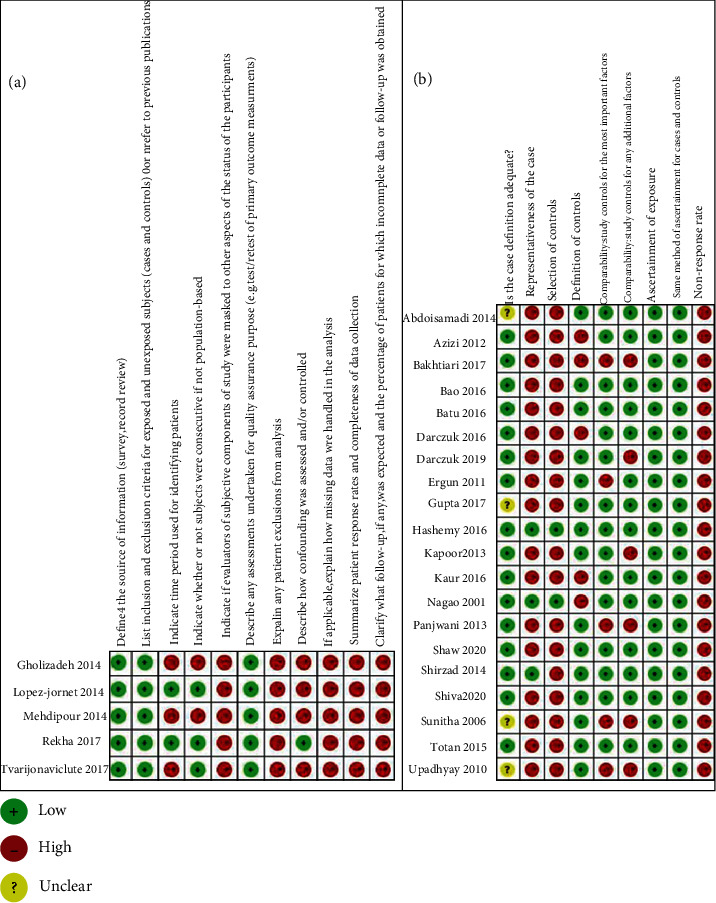
Bias risk of the included studies.

A total of 25 eligible studies were illustrated in detail in Table 1, which involved 849 OLP patients and 1,052 control subjects in the meta-analysis. Totally, 12 oxidative stress and antioxidant barrier markers were adopted in this meta-analysis. We present a summary of the findings of our meta-analyses in Table 2, which includes the number of studies/participants, the corresponding summary effect size, and *I*^2^ value for each meta-analysis. Figures 2–5 show forest plots of the oxidative stress, nitrosative stress, antioxidant barrier (enzymatic and nonenzymatic), and total redox status markers with the significant difference between OLP and healthy controls, respectively. All the studies had a cross-sectional and case-control design; patients were clinically/histopathologically evaluated for OLP. According to the assessment by the NOS and AHRQ checklist (Figure 6), we assigned a low quality to 3 studies and a moderate quality to 2 studies among the 5 cross-sectional studies. In 20 case-control studies, 3 were of high quality, 4 were of low quality, and the remaining 10 were of medium quality.

### 3.3. Oxidative Stress Markers in OLP

#### 3.3.1. Lipid Peroxidation Malondialdehyde (MDA)

Three studies [15, 28, 29] demonstrated significantly higher MDA in the serum/plasma from OLP patients while one study [30] mentioned no difference. Overall, MDA in the serum/plasma showed a significant increase in OLP (effect size 0.92, 95% CI 0.38, 1.46), with statistically significant heterogeneity (*P* = 0.04, *I*^2^ = 64%). MDA was measured in the saliva from OLP in nine studies and found to be markedly elevated in all nine (effect size 2.38, 95% CI 1.47, 3.29), with statistically significant heterogeneity (*P* < 0.00001, *I*^2^ = 94%) [16, 28–35].

#### 3.3.2. 8-Hydroxy-deoxy (8-OHdG)

For evidence of oxidative DNA/RNA damage, 8-OHdG in the saliva from OLP patients was evaluated in two studies (70 patients) and found to be increased in both under a random effects model (effect size 3.78, 95% CI 0.14, 7.42), with statistically significant heterogeneity (*P* < 0.00001, *I*^2^ = 97%) [29, 33]. Totan et al. also evaluated 8-OHdG in the serum and found a significant increase in OLP patients [29].

#### 3.3.3. Advanced Oxidation and Products (AOPP)

Only two studies (38 patients) evaluated AOPP levels in the saliva from patients with OLP under a fixed effects model; both showed a significant increase (effect size 1.00, 95% CI 0.54, 1.45), with no significant heterogeneity (*P* = 0.48, *I*^2^ = 0%) [28, 36]. In a single study, increased AOPP was reported in the serum from OLP patients [28].

### 3.4. Nitrosative Stress Markers in OLP

#### 3.4.1. Nitric Oxide (NO)

As for the NO level in saliva, all the four original studies (97 patients) demonstrated significantly higher NO level in OLP than in controls (effect size 3.25, 95% CI 1.11, 5.39) [36–39]. The same results were also presented in serum/plasma among two studies (effect size 1.07, 95% CI 0.60, 1.53) [39, 40]. The heterogeneity of NO in saliva was significant (*P* < 0.00001, *I*^2^ = 94%), while NO in serum/plasma had no significant heterogeneity (*P* = 0.31, *I*^2^ = 3%).

#### 3.4.2. Nitrite

Nitrite in saliva was evaluated in 2 studies, and contrast results were achieved. Lower nitrite in saliva was found by Sunitha et al. [41], whereas Tvarijonaviciute et al. showed a much higher level in OLP patients than in healthy controls [36]. Meta-analyzing data of nitrite in saliva revealed no significant difference in cases and controls.

### 3.5. Antioxidant Barrier in OLP

#### 3.5.1. Enzymatic Antioxidant

*(1) Glutathione Peroxidase (GPx)*. In saliva, three studies all reported significantly lower GPx in OLP cases, while there was no significant association of GPx with OLP across the meta-analyses [29, 35, 42]. In addition, GPx in serum [29] (30 patients), thiol in serum [15] (22 patients), and GSH in saliva [32] (62 patients) were determined in the single study, and decreased levels were found.

#### 3.5.2. Nonenzymatic Antioxidant

*(1) Uric Acid (UA)*. UA in plasma/serum was investigated in three studies and found to be markedly decreased in OLP compared to controls (effect size -1.19, 95% CI -1.83, -0.54), with statistically significant heterogeneity (*P* = 0.008, *I*^2^ = 80%) [29, 43, 44]. Four studies reported lower salivary UA in OLP cases [29, 42, 44, 45], while one single study did not report any statistically significant difference between OLP and controls [35]. The meta-analysis for UA assessed in saliva was -2.65 (95% CI -4.20, -1.09), with statistically significant heterogeneity (*P* < 0.00001, *I*^2^ = 97%).

*(2) Zinc (Zn)*. Contrasting data were reported from two studies on Zn levels in serum/plasma. Bao et al. showed a much lower Zn in the saliva of OLP patients [46], while the results were opposite in the other study by Gholizadeh et al. [47]. The meta-analysis for Zn in serum/plasma showed no significant difference between OLP and controls. Only one study evaluated Zn in saliva and showed no significant difference between OLP and healthy controls [48].

*(3) Vitamins*. Concerning vitamin A, one study with 36 OLP patients did not observe a difference while a large study with 62 OLP patients detected significantly higher vitamin A in saliva [26, 49]. As for vitamin C, both of two studies (76 patients) were conducted showing lower vitamin C in the saliva from OLP [31, 33]. In the case of vitamin E, two studies [31, 33] (76 patients) observed decreased vitamin E levels in saliva while a single study [49] (62 patients) detected no significant difference between patients with OLP and controls. Overall, the levels of vitamin C in saliva had a significant decrease in OLP (effect size -2.03, 95% CI -3.16, -0.89), with statistically significant heterogeneity (*P* = 0.005, *I*^2^ = 87%), while there were no significant differences of salivary vitamin E and vitamin A between OLP and controls.

#### 3.5.3. Others

Antioxidant nutrients and trace elements counteract free radical damage and thereby protect cell membranes against lipid peroxidation. Magnesium (Mg), copper (Cu), vitamin B12, folic acid, Tyr, TP, thiol, cryptoxanthin, lycopene, and nitrite were only investigated in a single study. Nagao et al. studied seven serum antioxidant micronutrients in 62 patients with OLP and 248 healthy control subjects, finding higher levels only of retinol (vitamin A) among the OLP patients; no significant differences were found for the other micronutrients, such as *α*-tocopherol, zeaxanthin and lutein, cryptoxanthin, lycopene, *α*-carotene, and *β*-Carotene [49]. Rezazadeh et al. studied five trace elements in 40 patients with OLP and 40 healthy control subjects, finding higher levels only of magnesium (Mg) among the OLP patients; no significant differences were found for the other trace elements, including calcium (Ca), iron (Fe), zinc (Zn), and copper (Cu) [48].

### 3.6. Total Redox Status

#### 3.6.1. Total Antioxidant Capacity (TAC)

Seven independent publications (267 patients) were measured on salivary TAC levels and found increased levels of TAC in six [16, 29, 31, 32, 34, 50], while there was no significant difference in one study [30]. Overall, TAC in saliva showed a significant decrease in OLP (effect size -2.03, 95% CI -3.03, -1.03), with statistically significant heterogeneity (*P* < 0.00001, *I*^2^ = 95%). TAC in the plasma/serum from OLP patients was assessed in four studies (121 patients) and showed a significant decrease in all four (effect size -2.87, 95% CI -4.56, -1.19), with statistically significant heterogeneity (*P* < 0.00001, *I*^2^ = 95%) [15, 29, 30, 50].

### 3.7. Subgroup Analysis

Subgroup analyses in salivary TAC and MDA studies were performed as categorized by race (Asian vs. European). The effect sizes are shown in Tables 3 and 4. From the results, salivary MDA studies showed a significantly larger effect size in Asians than in Europeans (*P* = 0.008). Subgroup analyses in salivary TAC studies showed no significant difference between Asians and Europeans (*P* = 0.42).

### 3.8. Sensitivity Analysis

The method of excluding the studies one by one was adopted to find if there was any research affecting the stability. The results of salivary TAC and MDA are shown in Tables 5 and 6. As shown in the tables, after excluding each single study, the salivary TAC and MDA were not significantly influenced and the *I*^2^ also did not change remarkably.

## 4. Discussion

Our study was aimed at clarifying and quantifying the oxidative stress and antioxidant markers in the saliva and serum/plasma of OLP. The reviewed data and the results of our meta-analyses point to a role for significantly increased oxidative stress indicators and decreased antioxidant markers in individuals with OLP compared to healthy controls.

**Figure 7:**
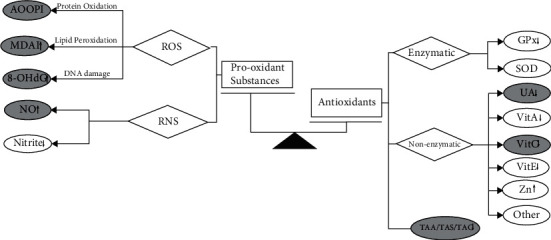
Schematic representation of the oxidative stress and antioxidant markers in oral lichen planus. Black arrows: markers with statistical differences in meta-analysis. Grey arrows: trend but no statistical differences. Unmarked: reported by a single study. Abbreviations: ROS = reactive oxygen species; RNS = reactive nitrogen species; MDA = malondialdehyde; AOOP = advanced oxidation protein product; 8-OHdG = 8-hydroxy-deoxyguanosine; NO = nitric oxide; GPx = glutathione peroxidase; SOD = superoxide dismutase; UA = uric acid; Vit = vitamin; Zn = zinc; TAC = total antioxidative capacity.

Oxidative stress is caused by an imbalance between prooxidant substances (such as reactive oxygen species (ROS) and reactive nitrogen species (RNS)) and the ability of the antioxidant system (enzymatic or nonenzymatic antioxidants) [51–54] (Figure 7). ROS are generated from multiple sources, both endogenous and exogenous. The oral cavity is a critical site for oxidative stress, and exogenous sources include oral tissue exposure to thermal, chemical, and microbial stimuli. Many behavioral factors are also conducive to the accumulation of exogenous ROS (smoking, alcohol use, and chewing betel nuts) and are also related to malignant transformation of OLP [55]. Endogenous sources refer to chronic or acute infections in oral, such as periodontitis and OLP [56]. Inflammatory cells are known to produce large amounts of ROS, and ROS in turn amplifies the inflammatory response [50].

ROS causes oxidative damage to the tissues via multiple mechanisms, including DNA damage, lipid peroxidation (LPO) damage, and protein oxidation [57–59]. 8-OHdG, both the most abundant and most investigated biomarker of oxidant-induced DNA damage, has mutagenic properties and is a risk factor for the development of cancer [60]. Although only two studies reported 8-OHdG, both of them found higher 8-OHdG in the saliva and serum from OLP patients [29, 33]. ROS has a short half-life, making direct detection difficult. Measurement of secondary products such as LPO is suggested as a feasible option to evaluate oxidative damage. The most studied marker in the saliva of OLP patients was MDA, which is an end product of LPO and can reflect the degree of cellular damage [50, 56, 61]. From our meta-analysis, we found the level of MDA markedly increased in both the saliva and serum/plasma of OLP patients. However, controversy arises in the use of MDA levels as optimal representation for oxidative stress as it could be a nonspecific product of LPO [62]. AOPP is a cross-linking protein product modified by the interaction of free radicals and a novel marker of oxidant-mediated protein damage [63]. Our meta-analysis showed higher salivary AOOP in OLP cases than in healthy controls [28, 36]. Consistent with other reviews, we also found that the level of NO in OLP patients was higher than that in healthy controls [19, 64]. In recent years, a growing number of studies have confirmed that NO could be involved in OLP pathogenesis [65, 66]. The reason is that it is a free radical gas acting as a “double-edged sword.” Low levels of NO are associated with hemostatic actions, while high amounts of NO can affect the ability of cells to kill bacteria, viruses, and protozoans as well as tumor cells and then participate in inflammatory and immunological disorders [40, 66, 67].

Moreover, in our meta-analysis, lower levels of antioxidants were found in OLP patients, possibly reflecting a weakness of the defense against oxidative damage in them. TAC as an integrated parameter may serve as a better marker instead of examining individual antioxidants [68–70]. In our report, the total antioxidative activity/status/capacity (TAA/TAS/TAC) was found to be decreased in patients with OLP, which may suggest that the antioxidant defense system was inhibited. UA is an important antioxidant in saliva and plasma with the free radical scavenging capacity [71, 72]. Our research showed that the level of UA markedly decreased in both the saliva and serum/plasma of OLP patients. Vitamin is a crucial factor in proper innate and acquired immune responses, which triggers the expression of the genes encoding the proteins involved in immune response [18]. Vit A and E inhibit the lipid peroxidation of the cell membrane, whereas Vit C functions as a cofactor for many enzymes. Besides, Vit C also helps reproduce Vit E [73]. Some researchers considered vitamins C and E suitable biomarkers to predict OLP [74]. Our study on Vit C in saliva showed a significantly lower level in OLP patients than in controls, while there was no significant difference in Vit A and E. However, considering the poor specificity of mechanisms and the strong dependency on diet limits, we think that vitamins could not be regarded as biomarkers of OLP. Moreover, some studies found that certain trace elements (Mg, Zn, etc.) may play an important role in regenerative processes against oxidative stress products in the tissues [48]. But other studies reached the opposite conclusion [49], and further research is needed in the future.

Overall, different studies in our systematic review suggested an increased oxidative stress and a decrease in antioxidant levels in OLP, thus proving that oxidative stress plays an important role in its pathophysiology. Although the etiology of OLP is unknown, it is characterized by subepithelial infiltration of T lymphocytes (CD4^+^ and especially CD8^+^ cells) [75]. It is believed that the generation of ROS may play a role in T cell immune response and then modify and cause dysregulation of immune functions by changing the balance of Th1/Th2 cytokines and increasing Th2 response [3, 76]. Additionally, excessive reactive oxygen species (ROS) may be also correlated with a malignant transformation of OLP. The resulting elevation of oxidative stress can lead to DNA damage, protein oxidation, and lipid peroxidation. These results coupled with lack of cellular repair processes have been shown to be associated with mutation-induced carcinogenesis [53, 63, 77].

We pooled only measurements carried out on the same biological sample (saliva or plasma/serum), but the heterogeneity of most studies was high other than the studies on NO in serum/plasma (*P* = 0.31, *I*^2^ = 3%) and AOOP in saliva (*P* = 0.48, *I*^2^ = 0%). As to the studies whose components may contribute to heterogeneity, we conducted subgroup and sensitivity analyses for salivary TAC and MDA. By subgroup analysis, we found that salivary MDA in Asians was significantly higher than that in Europeans (*P* = 0.008), while no racial difference was noted in salivary TAC (*P* = 0.42). The sensitive analysis showed that after excluding each single study, the salivary TAC and MDA and *I*^2^ were not markedly influenced. Thus, the robustness of the results was proven. We investigated further possible causes of heterogeneity among studies (e.g., host factor, sample collection, assay methods, and others). First, in terms of host factor, the individual patient characteristics varied greatly between studies (age, gender, race, body mass index (BMI), and smoking), and the factor of antioxidant supplementation and diet might both influence biomarker levels. The type, severity, and location of OLP could cause heterogeneity. Some studies focused on patients diagnosed with erosive OLP, while others did not divide OLP into different forms. The lower concentration of antioxidant defense markers and higher levels of oxidative stress markers were found in the erosive form as compared to other forms of OLP [32, 42]. The more serious condition of oxidative stress in patients with erosive OLP seemed to reflect more intense inflammation and potential capacity for malignant transformation. The levels of these markers were associated with OLP lesion severity [28]. However, all included studies were observational and at a significant risk of bias and confounding. The limited number of the studies included made it unclear whether publication bias could be a factor in these markers. Such biases can be resolved by increasing the number of included articles in future research. The imbalance between oxidant and antioxidant systems also plays a role in other oral diseases, including recurrent aphthous stomatitis (RAS) and periodontitis [78, 79]. Not all authors gave clear details on these points. Finally, different assay methods, testing equipment, sample collection status, and reagent kits in the included studies could cause measurement bias.

The strengths of this meta-analysis lie in our extensive and comprehensive search to identify all studies on the association of oxidative stress and antioxidant markers with OLP, rigorous evaluation and one-by-one analysis, and further statistical processing of the data through quantitative synthesis to reach comprehensive conclusions. Meanwhile, a relatively objective evaluation of oxidative stress and antioxidant markers in OLP could be performed by analyzing the heterogeneity of different studies.

Our meta-analysis showed that oxidative stress and antioxidant markers may be the potential biomarkers to diagnose OLP. Increase in oxidants and decrease in antioxidants might be an indicator of OLP occurrence. As the oral cavity is the start of the digestion system, saliva acts as the first line of defense against OS with abundant antioxidants, such as UA, albumin, and ascorbic acid [80]. We found that the markers in saliva and blood have the same variation tendency. Similar to our study, Ergun et al. showed a significant correlation between oxidant and antioxidant salivary and serum levels [30]. These results suggest that saliva can be considered a reliable medium in assessing oxidative stress levels, which makes it possible to diagnose OLP noninvasively. As a better substitute for blood and urine samples, saliva can serve as a diagnostic fluid and presents many advantages: it is noninvasive, safe, painless, and easy to be collected [35]. In addition, salivary OS markers represent the state of local oral oxidative stress, which can better reflect the real situation of local oral microenvironment. However, using saliva to measure oxidative stress in OLP also has some limitations. There is still a lack of unified and standardized processes for saliva collection time, and whether it needs to be stimulated or centrifuged after sampling, its storage temperature and time, and analysis methods all remain elusive [31].

This meta-analysis has several limitations. Firstly, the number of included studies on each marker with high heterogeneity is limited (*n* < 10), and more homogeneous studies on oxidative stress in OLP are needed. Secondly, the quality of the above literature was generally not high and had small sizes of the study groups. To further evaluate the role of oxidative and antioxidative stress markers in OLP, large and strict quality control studies in various regions are necessary. Thirdly, thus far, most published studies have assessed oxidant-antioxidant status in patients with OLP in comparison with healthy individuals and the correlation between the markers and the degree of OLP lesions. However, the diagnostic utility of salivary redox biomarkers has not been truly validated in OLP diagnosis. Estimation of specificity, sensitivity, predictive values, ROC analysis or cluster analysis, etc. has not been reported when attempting to use biomarkers as a diagnostic/prognostic markers in OLP. Further studies in this direction will help to find a reliable and unambiguous diagnostic or prognostic marker among the OS and antioxidant markers, which could be used as a therapeutic target in clinical practice. Last but not least, although the association between OLP and OS is well established and the patients with increased free radicals in serum and saliva and decreased activity of antioxidants support this thesis, it is not clear whether OS is the cause or a result of OLP. Further studies are required to investigate molecular mechanisms underlying the etiology of these pathologies and understand in depth the potential role of OS in OLP.

## 5. Conclusions

In conclusion, studies on saliva and plasma/serum biomarkers related to oxidative stress and antioxidants in OLP patients provide evidence suggestive of a significant increase in NO and MDA in saliva and plasma/serum and 8-OHdG and AOOP in saliva, as well as a significant decrease in TAC and UA in saliva and plasma/serum and Vit C in saliva. To sum up, NO, 8-OHdG, AOOP, TAC, and UA may be the potential biomarkers for measuring the effect imbalance of antioxidant-oxidative stress in OLP patients. Nevertheless, there are only a limited number of studies on some markers and with high heterogeneity; more homogeneous studies on oxidative stress in OLP are needed. Moreover, further studies are also needed, including how to explain the link by examining the underlying mechanisms and whether antioxidant therapy could be developed as a new significant therapy for decreasing inflammation and producing positive, long-term effects on OLP.

## Figures and Tables

**Table 1:** Description of the studies comparing oxidative/antioxidative markers in patients with OLP and controls.

			Sample size (males/females)		Age (years; mean ± SD or median [minimum–maximum])					
Author (publication year)	Country	Study design	Case	Control	Case	Control	OLP characteristics	Biomarkers of interest	Biological specimen	Quality score
Abdolsamadi (2014)	Iran	CC	36 (14/22)	36 (15/21)	43.1 ± 9.6	40.1 ± 6.1	Erosive OLP	TAC, MDA, Vit A, Vit E, Vit C	Saliva	5
Azizi (2012)	Iran	CC	48 (14/34)	44 (10/34)	40.2 ± 4.9	42.2 ± 4.9	Erosive OLP	TAC	Saliva, plasma	5
Bakhtiari (2017)	Iran	CC	25 (10/15)	30 (10/20)	46 ± 2.33	36 ± 1.18	Reticular (*n* = 7), erosive (*n* = 11), atrophic (*n* = 4) and ulcerative (*n* = 3)	UA	Saliva	3
Bao (2016)	China	CC	57 (13/44)	115 (43/72)	52.47 ± 15.13	53.10 ± 15.59	—	Zn	Serum	7
Batu (2016)	Turkey	CC	18 (5/13)	18 (9/9)	50.67 ± 12.39	49.22 ± 11.11	Reticular (*n* = 12), papular (*n* = 3), plaque-like (*n* = 2), erosive (*n* = 3)	MDA, SA, AOPP	Saliva, serum	6
Darczuk (2019)	Poland	CC	40 (20/20)	40 (20/20)	—	—	Reticular OLP (*N* = 26), erosive OLP (*N* = 14)	Tyr, UA, GPx	Saliva	5
Darczuk (2016)	Poland	CC	62 (30/32)	30 (10/20)	42.53 ± 10.69	42.12 ± 12.22	Reticular form of lichen planus (*n* = 31), erosive forms (*n* = 31)	GSH, TAC, TBARS	Saliva	5
Ergun (2011)	Turkey.	CC	21 (11/10)	20 (11/9)	54.7 ± 9.2	33.7 ± 6.9		TAC, MDA, TP	Saliva	5
Gholizadeh (2014)	Iran	CS	44	44	—	—	Erosive, nonerosive	Zinc	Serum	3
Gupta (2017)	India	CC	39 (14/25)	39 (14/25)	—	—	Erosive subtype 43.6%, atrophic 17.9%, plaque-like 7.7%, papular 2.6%, and bullae 2.6%	UA	Serum	5
Hashemy (2016)	Iran	CC	25 (8/17)	23 (7/16)	46.48 ± 11.08	43.70 ± 12.23	Erosive OLP, keratotic OLP	MDA, TAC	Serum	8
Kapoor (2013)	India	CC	25	25	[20–45]	[20–45]	Reticular type of OLP (*n* = 12), erosive (*n* = 4), plaque (*n* = 4), atrophic (*n* = 5)	NO	Saliva	5
Kaur (2016)	Belgium	CC	40 (20/20)	40 (20/20)	49 ± 5.8	48.9 ± 7.0	—	8-OHdG, MDA, vitamin E, vitamin C	Saliva	5
Lopez-Jornet (2014)	Spain	CS	40 (9/31)	30 (6/24)	60 ± 12.6	57 ± 10.8	Reticular-papular (*n* = 36), atrophic-erosive (*n* = 4)	TAC, MDA	Saliva	5
Mehdipour (2014)	Iran	CS	20	20	34.1	35.6	—	NO	Serum	3
Nagao (2001)	Japan	CC	62 (15/47)	248 (60/188)	60.6 ± 9.2	60.7 ± 9.1	Reticular (*n* = 58), atrophic and erosive (*n* = 4)	Retinol, *α*-tocopherol, zeaxanthin & lutein, cryptoxanthin, lycopene, *α*-carotene, *β*-carotene, total cholesterol	Serum	7
Panjwani (2013)	India		30	30	—	—	Reticular lichen planus (*n* = 27), erosive (*n* = 3)	NO		4
Rekha (2017)	India	CS	30 (13/17)	30 (15/15)	42.033 ± 1.57	45.17 ± 2.25	—	SOD, MDA, GPx, UA	Saliva	6
Shaw (2020)	India	CC	43 (14/29)	42 (15/27)	45.95 ± 15.10	44.24 ± 15.47	—	UA	Saliva, serum	6
Shirzad (2014)	Iran	CC	30 (4/26)	30 (4/26)	44.37 ± 8.16	44.77 ± 8.61	Erosive (*n* = 29), plaque (*n* = 1)	TAC, MDA	Saliva	7
Shiva (2020)	Iran	CC	22 (10/12)	22 (9/13)	48.7 ± 9.2	43.7 ± 6.9	—	NO, CRP	Saliva	6
								NO, CRP	Serum	
Sunitha (2006)	India	CC	20 (8/12)	20 (8/12)	37.7	33.4	—	Nitrite	Saliva	3
Totan (2015)	Romania	CC	30 (15/15)	30 (20/10)	—	—	—	8-OHdG, MDA, UA, TAC, GPx	Saliva, serum	6
Tvarijonaviciute (2017)	Spain	CS	20 (0/20)	31 (11/20)	57.5 [37.0–75.0]	33.0 [18.0–67.0]	—	TEAC1, TEAC2, CUPRAC, FRAP, NO, nitrates, nitrites, TEA, AOOP, ROS	Saliva	4
Upadhyay (2010)	India	CC	22	15 (8/7)	47	43	—	Thiol, MDA, TAA	Serum	3

CC = case control; CS = cross-sectional; TAC = total antioxidant capacity; MDA = malondialdehyde; Vit = vitamin; UA = uric acid; Zn = zinc; SA = sialic acid; AOOP = advanced oxidation protein product; GPx = glutathione peroxidase; GSH = glutathione; TBARS = thiobarbituric acid reactive substances; TAC = total antioxidant capacity; TP = total protein; NO = nitric oxide; SOD = superoxide dismutase; CRP=C-reactive protein.

**Table 2:** Meta-analyses of comparisons of oxidative/antioxidative markers between OLP patients and controls.

				Effect size (OLP vs. controls)			Heterogeneity			
Marker (source)	# of studies	OLP, *N*	Control, *N*	SMD (95% CI)	*Z* value	*P* value^∗^	Chi^2^	df	*P* value	*I*^2^ (%)
NO (saliva)	4	97	108	3.25 (1.11, 5.39)	2.98	*P* = 0.003	86.4	3	*P* < 0.00001	97
NO (serum/plasma)	2	42	42	1.07 (0.60, 1.53)	4.47	*P* < 0.00001	1.03	1	*P* = 0.31	3
MDA (saliva)	9	307	264	2.38 (1.47, 3.29)	5.13	*P* < 0.00001	138.81	8	*P* < 0.00001	94
MDA (serum/plasma)	4	91	83	0.92 (0.38, 1.46)	3.34	*P* = 0.0008	8.37	3	*P* = 0.04	64
8-OHdG (saliva)	2	70	70	3.78 (0.14, 7.42)	2.04	*P* = 0.04	38.24	1	*P* < 0.00001	97
AOOP (saliva)	2	39	49	1.00 (0.54, 1.45)	4.31	*P* < 0.00001	0.50	1	*P* = 0.48	0
TAC (saliva)	7	267	220	-2.03 (-3.03, -1.03)	3.98	*P* < 0.0001	115.29	6	*P* < 0.00001	95
TAC (serum/plasma)	4	121	109	-2.87 (-4.56, -1.19)	3.34	*P* = 0.0008	57.84	3	*P* < 0.00001	95
Vit C (saliva)	2	76	76	-2.03 (-3.16, -0.89)	3.5	*P* = 0.0005	7.87	1	*P* = 0.005	87
UA (saliva)	5	168	173	-2.65 (-4.20, -1.09)	3.33	*P* = 0.00009	127.97	4	*P* < 0.00001	97
UA (serum/plasma)	3	112	111	-1.19 (-1.83, -0.54)	3.61	*P* = 0.0003	9.77	2	*P* = 0.008	80
Vit A (saliva)	2	98	284	-0.86 (-3.24, 1.52)	0.71	*P* = 0.48	54.52	1	*P* < 0.00001	98
Zn (serum/plasma)	2	101	159	0.21 (-1.73, 2.16)	0.22	*P* = 0.83	47.99	1	*P* < 0.00001	98
GPx (saliva)	3	100	100	-1.34 (-2.81, 0.13)	1.78	*P* = 0.07	42.02	2	*P* < 0.00001	95
Vit E (saliva)	3	138	324	-1.53 (-3.41, 0.34)	1.60	*P* = 0.11	92.75	2	*P* < 0.00001	98
Nitrite (saliva)	2	40	51	-0.23 (-2.81, 2.35)	0.17	*P* = 0.86	30.43	1	*P* < 0.00001	97

TAC = total antioxidant capacity; Vit C = vitamin C; UA = uric acid; NO = nitric oxide; MDA = malondialdehyde; AOOP = advanced oxidation protein product; Vit A = vitamin A; Zn = zinc; GPx = glutathione peroxidase; Vit E = vitamin E.

**Table 3:** Subgroup analysis of TAC (saliva).

Subgroup		*N*	SMD (95% CI)	*P-*heterogeneity	*I*^2^ (%)	*P*-interaction
Race	Asian	4	-2.41 (-3.95, -0.86)	<0.00001	98	0.42
	European	3	-1.55 (-2.93, -0.17)	<0.00001	98	

**Table 4:** Subgroup analysis of MDA (saliva).

Subgroup		*N*	SMD (95% CI)	*P-*heterogeneity	*I*^2^ (%)	*P*-interaction
Race	Asian	5	1.30 (0.76, 1.85)	<0.00001	83	0.008
	European	4	0.92 (0.62, 1.21)	<0.00001	100	

**Table 5:** Sensitivity analysis of TAC (saliva) using the method of eliminating literature one by one.

Deleted article	*I*^2^ (%)	*P*	SMD (95% CI)
Abdolsamadi	93	<0.00001	-1.67 (-2.59, -0.74)
Azizi	95	<0.00001	-2.01 (-3.20, -0.81)
Darczuk	96	<0.00001	-2.10 (-3.34, -0.87)
Ergun	95	<0.00001	-2.33 (-3.39, -1.27)
Lopez-Jornet	93	<0.00001	-2.33 (-3.31, -1.35)
Shirzad	95	<0.00001	-1.89 (-2.98, -0.80)
Totan	95	<0.00001	-1.91 (-3.01, -0.80)

**Table 6:** Sensitivity analysis of MDA (saliva) using the method of eliminating literature one by one.

Deleted article	*I*^2^ (%)	*P*	SMD (95% CI)
Abdolsamadi	95	<0.00001	2.54 (1.50, 3.58)
Batu	94	<0.00001	2.61 (1.64, 3.58)
Darczuk	95	<0.00001	2.35 (1.33, 3.37)
Ergun	95	<0.00001	2.58 (1.59, 3.57)
Kaur	90	<0.00001	1.87 (1.18, 2.55)
Lopez-Jornet	94	<0.00001	2.16 (1.26, 3.06)
Rekha	95	<0.00001	2.39 (1.37, 3.42)
Shirzad	95	<0.00001	2.51 (1.47, 3.55)
Totan	95	<0.00001	2.47 (1.43, 3.52)

## Data Availability

The data used to support the findings of this study are included within the article.
